# Hypoglycemic effects of *Trichosanthes kirilowii* and its protein constituent in diabetic mice: the involvement of insulin receptor pathway

**DOI:** 10.1186/s12906-017-1578-6

**Published:** 2017-01-18

**Authors:** Hsin-Yi Lo, Tsai-Chung Li, Tse-Yen Yang, Chia-Cheng Li, Jen-Huai Chiang, Chien-Yun Hsiang, Tin-Yun Ho

**Affiliations:** 1 Graduate Institute of Chinese Medicine, China Medical University, 91 Hsueh-Shih Road, Taichung, 40402 Taiwan; 2 Graduate Institute of Biostatistics, China Medical University, Taichung, 40402 Taiwan; 3 Molecular and Genomic Epidemiology Center, China Medical University Hospital, Taichung, 40447 Taiwan; 4 Management Office for Health Data, China Medical University Hospital, Taichung, 40447 Taiwan; 5 Department of Microbiology, China Medical University, 91 Hsueh-Shih Road, Taichung, 40402 Taiwan; 6 Department of Health and Nutrition Biotechnology, Asia University, Taichung, 41354 Taiwan

**Keywords:** *Trichosanthes kirilowii*, Diabetes, Hypoglycemia, Insulin receptor

## Abstract

**Background:**

Diabetes is a serious chronic metabolic disorder. *Trichosanthes kirilowii* Maxim. (TK) is traditionally used for the treatment of diabetes in traditional Chinese medicine (TCM). However, the clinical application of TK on diabetic patients and the hypoglycemic efficacies of TK are still unclear.

**Methods:**

A retrospective cohort study was conducted to analyze the usage of Chinese herbs in patients with type 2 diabetes in Taiwan. Glucose tolerance test was performed to analyze the hypoglycemic effect of TK. Proteomic approach was performed to identify the protein constituents of TK. Insulin receptor (IR) kinase activity assay and glucose tolerance tests in diabetic mice were further used to elucidate the hypoglycemic mechanisms and efficacies of TK.

**Results:**

By a retrospective cohort study, we found that TK was the most frequently used Chinese medicinal herb in type 2 diabetic patients in Taiwan. Oral administration of aqueous extract of TK displayed hypoglycemic effects in a dose-dependent manner in mice. An abundant novel TK protein (TKP) was further identified by proteomic approach. TKP interacted with IR by docking analysis and activated the kinase activity of IR. In addition, TKP enhanced the clearance of glucose in diabetic mice in a dose-dependent manner.

**Conclusions:**

In conclusion, this study applied a bed-to-bench approach to elucidate the hypoglycemic efficacies and mechanisms of TK on clinical usage. In addition, we newly identified a hypoglycemic protein TKP from TK. Our findings might provide a reasonable explanation of TK on the treatment of diabetes in TCM.

## Background

Diabetes mellitus is a commonly metabolic syndrome that occurs because either the beta islet of pancreas does not produce enough insulin or the body cannot effectively utilize insulin [1, 2]. The prevalence of diabetes continues to grow, with diagnosed diabetes now reaching 415 million worldwide. About 2.5 to 15% of medical expenditures in many countries are attributed to diabetes [3]. There are many drugs currently used in the treatment of diabetes. However, some reports indicate that treatment with synthetic drugs is responsible for various adverse effects, such as hypoglycemia and gastrointestinal problems [4]. Therefore, the approach of better agents from traditional Chinese medicine (TCM) or natural products has been gaining a significant importance now, even in coming years [5].

TCM has been practiced for thousands of years in China and the Far East, and plays a major role in the health care [5–7]. Since ancient times, several Chinese herbal formulae as well as Chinese medicinal herbs have been commonly used in patients with Xiao Ke (消渴), a diabetic condition characterized by persistent thirst and hunger, copious urination, and weight loss. For example, Chu-Yeh-Shih-Kao-Tang (CYSKT) is a TCM formula that is composed of bamboo leaves, gypsum, pinellia rhizome, ginseng root, licorice root, rice, and ophiopogon tuber. It is traditionally used for the treatment of respiratory diseases and diabetes in China. Our previous study indicated that CYSKT significantly reduces glycated hemoglobin A1c values in diabetic patients in Taiwan. It also reduces fasting blood glucose levels and stimulates blood glucose clearance in diabetic mice via affecting insulin signaling pathway [8]. Danzhi Jiangtang Capsule, a Chinese medicinal formula consisting of cortex moutan, heterophylly falsestarwort root, unprocessed rehmannia root, oriental waterplantain rhizome, dodder seed and leech, has been used for treatment of diabetes for many years. Recent study showed that Danzhi Jiangtang Capsule attenuates streptozotocin (STZ)-induced type 1 diabetes in rats via the suppression of pancreatic beta cell apoptosis [9].

*Trichosanthes kirilowii* Maxim. (TK) is a member of family *Cucurbitaceae*. Trichosanthes root, also named as gualou or Tian-Hua Fen, is firstly described in Tujing Bencao (Illustrated Classics of Materia Medica) 950 year ago. It is traditionally used for the treatment of diabetes and its complications in China, Taiwan, and Eastern Asia [10, 11]. Previous study indicated that trichosanthes root and its glycan constituents exhibit hypoglycemic activities in normal or alloxan-induced hyperglycemic mice [12]. Lectins from TK also display hypoglycemic effects in alloxan-induced diabetic mice and stimulate the incorporation of D-[^3^H]glucose into lipids in isolated rat epididymal adipocytes [13, 14]. However, the clinical application of trichosanthes root on diabetic patients, and the hypoglycemic mechanisms of TK and its constituents are still unclear. To address these questions, we applied a bed-to-bench approach by surveying the usage of TK in clinics and analyzing the glucose clearance abilities of TK in mice. Two-dimensional electrophoresis (2-DE) coupled with liquid chromatography and tandem mass spectrometry (LC-MS/MS) was applied to identify the protein constituents of TK. Insulin receptor (IR) kinase activity assay and glucose tolerance tests in diabetic mice were further used to elucidate the hypoglycemic mechanisms and efficacies of TK.

## Methods

### Prescription pattern of TCM on diabetic patients in National Health Insurance system

A retrospective study was conducted using registration and claim datasets of the year 2002 from National Health Insurance Research Database (NHIRD), which covers claims of ambulatory care, inpatient services, dental services, and prescriptions from 99% of the overall population in Taiwan. The observed patients were identified from NHIRD by a principal diagnosis of diabetes (International Classification of Diseases, Nine Revision, Clinical Modification ICD-9-CM, 250 and 250.0). There were 774,367 patients diagnosed as type 2 diabetes in 2002. All patients with type 2 diabetes and TCM treatments were included. Patients’ records/information were anonymized and de-identified prior to analysis. This study was approved by Ethics Review Board of Chinese Medical University Hospital (Permit No. DMR97-IRB-272).

### Observation of TK usage in Longitudinal Health Insurance Database 2000 (LHID 2000)

LHID 2000 contains one million enrollees of all the original NHIRD, which was randomly sampled from Registry for Beneficiaries of the NHIRD during the period of 1996–2008. There are approximately 23.75 million individuals in this registry. The usage of TK in patients with endocrine, nutritional and metabolic diseases, and immunity disorders (ICD-9-CM, 240–279) in LHID 2000 was conducted a retrospective cohort study.

### Preparation of aqueous extract of TK (TKE)

Roots of TK were purchased from Sun-Ten Pharmaceutical Company (Taipei, Taiwan). The voucher specimens have been deposited in the Graduate Institute of Chinese Medicine, China Medical University. Trichosanthes roots were ground to fine powders using sample grinders. Powdered samples were extracted with phosphate-buffered saline (PBS) (137 mM NaCl, 1.4 mM KH_2_PO_4_, 4.3 mM Na_2_HPO_4_, 2.7 mM KCl, pH 7.2). The extracts were centrifuged at 15,000 g for 15 min, the lipid layer was removed, and the supernatant was then collected and lyophilized. The recovery amount of dried TKE was approximately 20–25 mg/g samples.

### Animal experiment and glucose tolerance tests

Five-week-old female BALB/c and male C57BL/6J mice were obtained from National Laboratory Animal Center (Taipei, Taiwan). Mice were maintained under a 12 h day-12 h night cycle and had free access to water and food. Mouse experiments were conducted under ethics approval from China Medical University Animal Care and Use Committee (Permit No. 104-75-N).

To induce type 1 diabetes, C57BL/6J mice were injected daily with 50 mg/kg STZ by an intraperitoneal route for five consecutive days. Fourteen to 16 days after final injection, fasting blood glucose levels were determined by a glucose oxidase method using a glucometer (ACCU-CHEK Advantage, Roche Diagnostics, Basel, Switzerland). To induce type 2 diabetes, C57BL/6J mice were fed with high-fat diet (TestDiet, St. Louis, MO, USA), in which 60% of energy was from fat. One week later, mice were intraperitoneally given with 100 mg/kg STZ and 240 mg/kg nicotinamide on days 0 and 2. Fasting blood glucose levels were determined on 30 days after challenge [15]. Mice with fasting blood glucose levels ≥230 mg/dL were selected and divided randomly. Glucose tolerance test was performed as described previously [16, 17]. Briefly, mice were starved overnight and TKE or TKP were then orally given 15 min before intraperitoneally injection of glucose solution (4 g/kg for normal mice and 1 g/kg for diabetic mice). Blood samples were collected from tails at 0, 30, 60, 90, 120, 180, or 240 min after glucose challenge. Glucose clearance was evaluated by calculating the area under the curve (AUC) of the glycemic profile.

### 2-DE and LC-MS/MS analysis of TKE

The protein composition of TKE was analyzed by 2-DE and LC-MS/MS as described previously [16]. Briefly, trichloroacetic acid-precipitated protein samples (200 μg) were applied to IPG strips (7 cm, pH 3-10). Isoelectric focusing was performed using a Protean IEF Cell (Bio-Rad, Hercules, CA, USA) by the following program: 0–250 V over 250 Vh, 250–4000 V over 4000 Vh, and 4000 Vh for 20,000 V. Focused IPG strips were then separated by sodium dodecyl sulfate-polyacrylamide gel (SDS-PAGE) on 15% acrylamide gels. Protein spots on the gels were visualized by Coomassie Brilliant Blue R-250.

For LC-MS/MS analysis, protein spots were excised from stained gels, in-gel digested by trypsin, and then identified using an Ultimate capillary LC system (LC Package, Amsterdam, The Netherlands) coupled to a QSTARXL quadrupole-time-of-flight mass spectrometer (Applied Biosystem/MDS Sciex, Foster City, CA, USA). MS/MS data were matched against NCBI Inr and Swiss-Prot using the MASCOT search program (http://www.matrixscience.com/).

### Homologous modeling and molecular docking

The structure of identified protein from TK was modeled using trypsin inhibitor from *Momordica charantia* (PDB code 1VBW) as a reference protein. PatchDock was used for the prediction of interaction between identified protein and IR (PDB code 3LOH).

### Cloning and purification of TK protein (TKP)

To clone the TKP cDNA, total RNA was extracted from TK, reverse transcribed by SuperScript® III, and amplified with P1 (5′- GATCAAGCTTATGTGTCAGGGGAAGTCGTCGTGGCCGCAG-3′) and M1 (5′-GATCGAGCTCTCAACCGATGGTGGGGGGGCGGGCGACGAT-3′) primers. The resulting 215-bp TKP cDNA fragment was inserted into *Hind*III and *Sac*I sites of pBluescript II KS (-) vector to create pBKS-TKP. DNA was sequenced on both strands of at least two repeats of cloned DNA fragments. The protein sequence of TKP has been deposited in GenBank (accession number: KP677558). TKP was purified by gel filtration as described previously [18]. The purity of TKP was approximately 95%, judged by SDS-PAGE.

### IR kinase activity assay

The binding of TKP to IR was measured by IR kinase activity assay. IR kinase activity assay was performed as described previously [17, 18]. Briefly, mixtures containing IR (Sigma, St. Louis, MO, USA) and various amounts of insulin or TKP in kinase buffer (25 mM HEPES, pH 7.6, 25 mM MgCl_2_, 100 μM ATP, 100 μM sodium orthovanadate, 2.5 mg/mL poly(Glu,Tyr), 25 μCi/mL [γ-^32^P]ATP) were incubated at 30 °C for 10 min and spotted on chromatography papers. Poly(Glu,Tyr) was precipitated on papers by soaking papers in 10% trichloroacetic acid solution, and the radioactivity incorporated into the precipitated poly(Glu,Tyr) was counted by scintillation counter.

### Statistical analysis

Continuous variables were presented as mean ± standard error. Category variables were estimated the statistical significance by one-way ANOVA and post hoc Bonferroni test using SPSS Statistics version 20 (IBM, Armonk, NY, USA). A *p* value less than 0.05 was considered as statistical significance.

## Results

### Evidence-cased Chinese medicinal herbs commonly used in diabetic patients in Taiwan

We conducted a retrospective cohort study to analyze the Chinese medicinal herbs commonly used among diabetic patients. The most frequently prescribed Chinese medicinal herb was TK (5.71%), followed by *Astragalus mongholicus* (4.76%), *Salvia miltiorrhizae* (4.66%), *Dioscoreae opposita* (4.18%), *Scrophularia ningpoensis* (2.78%), *Ophiopogonis japonicus* (2.68%), *Pueraria lobata* (2.40%), *Atractylodes lancea* (1.74%), *Dendrobium nobile* (1.73%), and *Rehmannia glutinosa* (1.49%) (Table 1).

**Table 1:** Top 10 Chinese medicinal herbs prescribed for type 2 diabetes patients in Taiwan

Chinese medicinal herbs (Chinese name)	*N* ^a^ (%)
*Trichosanthes kirilowii* Maxim., root (Tian Hua Fen)	42,013 (5.71)
*Astragalus mongholicus* Bunge, root (Huang Qi)	35,025 (4.76)
*Salvia miltiorrhizae* Bge, root (Dan Shen)	34,287 (4.66)
*Dioscoreae opposita* Thunb., root (Shan Yiao)	30,769 (4.18)
*Scrophularia ningpoensis* Hemsl., root (Xuan Shen)	20,421 (2.78)
*Ophiopogon japonicus* (Thunb.) Ker Gawl., tuber (Mai Men Dong)	19,744 (2.68)
*Pueraria lobata* (Willd.) Ohwi, root (Ge Gen)	17,641 (2.40)
*Atractylodes lancea* (Thunb.) DC., root (Cang Zhu)	12,803 (1.74)
*Dendrobium nobile* Lindl., stem (Shi Hu)	12,749 (1.73)
*Rehmannia glutinosa* Libosch, root (Di Huang)	10,948 (1.49)

^a^Total prescription number is 141,103

**Fig. 1:**
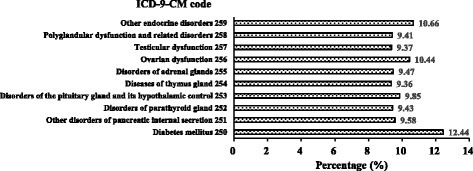
Analysis of the usage of TK in LHID 2000. Usage distributions (%) of TK in patients with endocrine disorders (ICD-9-CM codes 250–259)

We further analyzed the disease pattern that TK was frequently used for. By analyzing the LHID 2000, we found that 70,093 patients (15.83%) with endocrine, nutritional and metabolic diseases, and immunity disorders had treated with TK. By further analyzing the frequency of TK used among various endocrine disorders (ICD-9-CM, 250–259), we found that diabetes mellitus (ICD-9-CM, 250), other endocrine disorders (ICD-9-CM, 259), and ovarian dysfunction (ICD-9-CM, 256) were top three endocrine diseases that TK was used for. These findings suggested that the most common disease that TK was treated for was diabetes (Fig. 1). Moreover, TK was also the most frequently used Chinese medicinal herb in diabetic patients in Taiwan.

### TKE exhibited hypoglycemic effects with a biological gradient in BALB/c mice

**Fig. 2:**
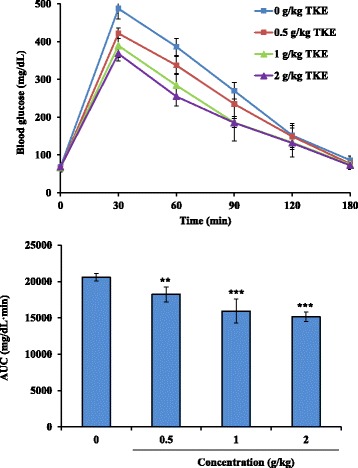
Hypoglycemic effects of TKE in mice. BALB/c mice were orally administered PBS, various amounts of TKE. Fifteen minutes later, glucose solution (4 g/kg) was administered intraperitoneally, blood samples were collected at the indicated time points, and blood glucose levels were measured by a glucometer. AUC of glycemic profile is shown at the *bottom*. Data are expressed as mean ± standard error (*n* = 5). **p < 0.01, ***p < 0.001, compared with mock

To analyze whether TK exhibited hypoglycemic effects, we prepared aqueous extract of TK and performed glucose tolerance assay in BALB/c mice. As shown in Fig. 2, fasting blood glucose levels of mice were approximately 70 mg/dL, and the blood glucose concentration reached a maximal level at 30 min after glucose challenge. Oral administration of TKE resulted in a more rapid clearance of glucose than that observed in mock group. Moreover, the hypoglycemic activity of TKE displayed a dose-dependent manner, and the inhibition reached 26.4 ± 3.19% at 2 g/kg. These data suggested that TKE exhibited hypoglycemic abilities in mice.

### Identification of protein constituents in TKE

**Fig. 3:**
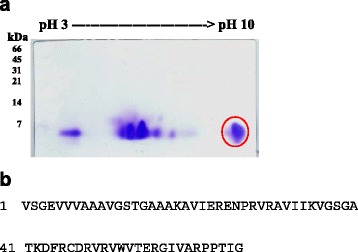
2-DE and LC-MS/MS analysis of protein constituents in TKE. **a** 2-DE. TKE was separated on pH 3-10 strips, followed by SDS-PAGE on 15% polyacrylamide gels. Proteins were visualized by Coomassie Brilliant Blue R-250. Protein size markers (in kDa) are shown on the *left*. Protein spot in *red circle* was excised and analyzed by LC-MS/MS. Photos are representative images of three independent experiments. **b** TKP sequences obtained by LC-MS/MS

Proteins are major components in the aqueous extract of plants. 2-DE coupled with LC-MS/MS analysis was therefore performed to evaluate the composition of protein constituents in TKE. As shown in Fig. 3a, TKE contained several low-molecular weight proteins and there was a visible and abundant low-molecular weight protein spot with an isoelectric point of 10 on the gels of TKE. The content of this protein spot in TKE was 35.21% (Fig. 3a, red circle).

We further excised this protein spot from gels and identified the protein by LC-MS/MS. By comparison with green plants (*Viridiplantae*) taxonomy, we found that there were no known proteins in database which were matched to the amino acid sequences of this protein spot in TKE. Therefore, we cloned and identified a novel protein TKP from TK based on the LC-MS/MS-defined amino acid sequences (Fig. 3b).

### TKP activated IR kinase activities

**Fig. 4:**
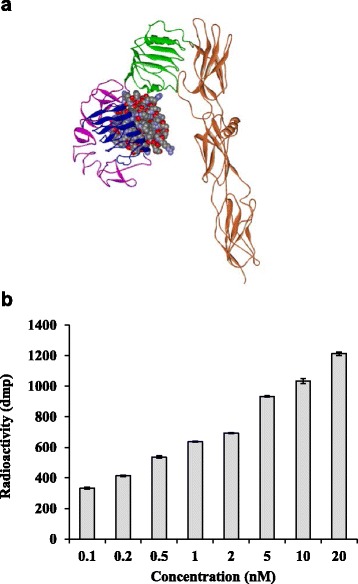
Interaction between TKP and IR. **a** Docking analysis. The IR ectodomain monomer is shown in a schematic presentation. Individual domains are colored as follows: L1, *blue*; CR, *pink*; L2, *green*; fibronectin type III domain, *orange*. TKP is shown in an atomic sphere representation. **b** IR kinase activity assay. Various amounts of TKP were incubated with IR in the presence of ^32^P-ATP and poly(Glu,Tyr). The radioactivity (dpm) incorporated into the precipitated poly(Glu,Tyr) was counted by a scintillation counter

Docking analysis was further performed to predict the interaction between TKP and IR. As shown in Fig. 4a, TKP directly docked into IR and fitted to the leucine-rich region L1 and cystein-rich region CR of IR. IR is a transmembrane protein that exhibits the tyrosine kinase activity. Binding of insulin to IR stimulates the intrinsic tyrosine kinase activity, leading to the autophosphorylation of IR. Therefore, we performed IR kinase activity assay to analyze whether TKP interacted with IR and activated IR kinase activities. As shown in Fig. 4b, the radioactivities incorporated into the precipitated poly(Glu,Tyr) were increased in the presence of TKP, and the increase displayed a dose-dependent manner. These data suggested that TKP was able to activate IR kinase activities.

### TKP stimulated the clearance of glucose in diabetic mice

**Fig. 5:**
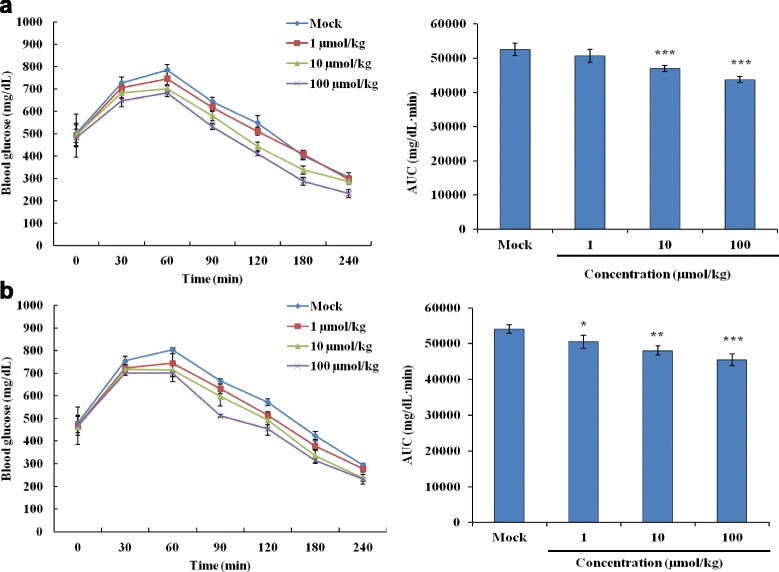
Hypoglycemic effects of TKP in diabetic mice. Type 1 (**a**) and type 2 (**b**) diabetic C57BL/6J mice were orally administered various amounts of TKP. Blood glucose levels were measured after 1 g/kg glucose challenge. AUC of glycemic profile is shown at the *right*. Data are expressed as mean ± standard error (*n* = 5). *p < 0.05, **p < 0.01, ***p < 0.001, compared with mock

To analyze whether TKP displayed hypoglycemic effects in diabetic C57BL/6J mice, we orally administered mice with various amounts of TKP. As shown in Fig. 5, the fasting blood glucose levels of type 1 and type 2 diabetic mice were approximately 500 mg/dL and 480 mg/dL, respectively. After glucose challenge, the levels of blood glucose reached to 800 mg/dL at 1 h. TKP stimulated the glucose clearance in both type 1 and type 2 diabetic mice, compared with mock. Moreover, the stimulation displayed a dose-dependent manner. These findings suggested that TKP might be the potent ingredient responsible for the blood glucose-modulating ability of TK.

## Discussion

TK has been widely used for treating cardiovascular, cerebrovascular, and respiratory diseases due to the clearance of heat, the dissipation of phlegm, the amelioration of chest stuffiness, and the regulation of flow of vital energy in TCM [10]. In combination with other Chinese medicinal herbs, TK is also used for cancer treatment [19]. Ni et al. (2015) reported that trichosanthes fruits inhibit non-small cell lung cancer cell growth through cell-cell and mitosis arrest [20]. In addition, triterpenoid-enriched extract of trichosanthes roots display anti-inflammatory activities in experimental acute and chronic inflammation models in rats [21]. In combination with other herbs, TK also displays an anti-arthritic efficacy in patients with osteoarthritis of the knee and anti-allergic inflammation in murine asthma model [22, 23]. TK is able to clear the heat, promote the production of body fluids, and resolve the swelling. Therefore, it has been traditionally prescribed for patients with diabetes, coughing, breast abscess, inflammation, and cancer-related symptoms in TCM.

TK is a member of family *Cucurbitaceae*. Plants of *Cucurbitaceae* family are cultivated throughout the world for use as nutritious vegetables as well as medications. Extracts of some gourds, such as *Momordica charantia*, *Cucurbita maxima* and *Cucumis sativus*, have been commonly used for the treatment of diabetes and related conditions among the indigenous populations of Asia, South America, India, and East Africa [24–26]. Animal and human studies also indicate the hypoglycemic effects of gourds. For example, extracts of *Cucumis sativus* seeds are effective on diminishing blood glucose levels and controlling the loss of body weight in STZ-induced diabetic rats through a mechanism similar to euglycemic agents [27]. Oral administration of pumpkin extract significantly decreases blood glucose levels in STZ-induced rats and diabetic patients via the increase of insulin secretion, the increase of β-cell mass, or the inhibition of α-amylase and α-glucosidase [28, 29]. In addition, extracts of *Momordica charantia* reduce blood glucose levels in diabetic rats and patients via the stimulation of translocation of glucose transporter 4, the promotion of β-cell recovery, and the inhibition of protein-tyrosine phosphatase 1B [30–32]. In this study, we found that TK was the most frequently used Chinese medicinal herb in diabetic patients in Taiwan. In addition, TKP displayed hypoglycemic effects in mice. These findings suggested that plants or herbs belonging to family *Cucurbitaceae* might commonly exhibit blood glucose-modulating abilities.

Several constituents with various pharmacological activities have been identified from TK. For example, trichosanthin is a 27-kDa ribosome inactivating protein that displays abortifacient, anti-viral, and immune-regulatory functions [33]. It also exhibits anti-cancer activities by the induction of apoptosis through G1 arrest, anti-telomerase effects, and anti-metastatic abilities [34, 35]. A serine protease with 46.62 kDa in TK fruits displays a potent anti-colorectal cancer activity by inducing apoptosis via phosphatidylinositol 3′-kinase/Akt-mediated mitochondria-dependent pathway [36]. Trichosans A, B, C, D, and E are glycans isolated from the water extract of trichosanthes roots. These glycans show hypoglycemic actions in normal mice, while trichosan A also exhibits a hypoglycemic activity in alloxan-induced hyperglycemic mice [12]. Saponins, flavonoids, triterpenes, and proteins have been identified as hypoglycemic components in gourds. For example, phenolic phytochemicals and protein-bound polysaccharides from fruits of *Cucurbita maxima* reduce blood glucose and improve glucose tolerance via the inhibitions of α-amylase and α-glucosidase [28, 29]. Bitter melon-derived triterpenoids activate AMP-activated protein kinase, increase glucose transporter 4 translocation to the plasma membrane in vitro, and improve glucose disposal in insulin-resistant models in vivo [37]. Polypeptide-p, M.Cy protein, MC6, and charantin from *Momordica charantia* show hypoglycemic effects in normal and diabetic mice [31, 38, 39]. In addition, an IR-binding protein in *Momordica charantia* binds to IR, triggers insulin signaling transduction, and stimulates the glucose clearance in vitro and in vivo [17, 18]. In the present study, we newly identified a novel TKP that exhibited hypoglycemic abilities in diabetic mice. Because of the abundance of TKP in the extract of TK, we speculated that TKP might be the potent hypoglycemic protein responsible for the blood glucose-modulating abilities of TK.

## Conclusion

In conclusion, TK is traditionally used for the treatment of diabetic patients in TCM. In this study, we found that TK is the most common used herb in type 2 diabetic patients in Taiwan. TKE exhibited hypoglycemic abilities in mice. TKP in TK interacted with IR, stimulated IR kinase activity, and enhanced the glucose clearance in diabetic mice. This is the first report applying a bed-to-bench approach to elucidate the hypoglycemic efficacies of TK. Our findings might provide a reasonable explanation of TK on the treatment of diabetes in TCM.

## Abbreviations

2-DE: Two-dimensional electrophoresis
AUC: Area under the curve
CYSKT: Chu-Yeh-Shih-Kao-Tang
ICD-9-CM: International Classification of Diseases, Nine Revision, Clinical Modification
IR: Insulin receptor
LC-MS/MS: Liquid chromatography and tandem mass spectrometry
LHID 2000: Longitudinal Health Insurance Database 2000
NHIRD: National Health Insurance Research Database
PBS: Phosphate-buffered saline
SDS-PAGE: Sodium dodecyl sulfate-polyacrylamide gel
STZ: Streptozotocin
TCM: Traditional Chinese medicine
TK: *Trichosanthes kirilowii* Maxim.
TKE: aqueous extract of TK
TKP: TK protein
